# The outcomes of cataract surgery in eyes with Fuchs uveitis

**DOI:** 10.1186/s12348-022-00320-3

**Published:** 2023-02-13

**Authors:** Yasemin Özdamar Erol, Kübra Özdemir Yalçınsoy, Pınar Özdal

**Affiliations:** Department of Uveal Diseases, University of Health Science, Ulucanlar Eye Education and Research Hospital, 06240 Ankara, Türkiye

**Keywords:** Cataract, Fuchs’ Uveitis, Intraocular lenses, Phacoemulsification, Inflammation

## Abstract

**Background:**

To evaluate clinical results in eyes undergoing phacoemulsification intraocular lens (IOL) implantation due to Fuchs^,^ uveitis (FU) related complicated cataract.

**Methods:**

Post-surgical outcomes of 56 eyes of 55 FU patients were evaluated retrospectively. Three groups were formed according to the IOL model: hydrophilic SAF6125 (Optima fold) acrylic, hydrophobic SN60AT (Alcon), and hydrophobic AAB00 (Abbott). Postoperative posterior capsular opacification (PCO) development and PCO development time, neodymium number: YAG laser posterior capsulotomy rate, giant cell (GC) deposition on the IOL, and the development time of GC deposits were compared among the groups. All patients were followed postoperatively on the 1^st^ day, 1^st^ week, 2^nd^ and 6^th^ weeks, and then at 3-month intervals.

**Results:**

The hydrophilic SAF6125 IOL was implanted in 10 eyes, hydrophobic SN60AT in 24 eyes and AAB00 IOL in 22 eyes. The mean postoperative follow-up time was 34.1 ± 30.1 (6-144) months. PCO developed in 7 eyes (70%) in the hydrophilic SAF6125 group, 17 eyes (70.8%) in the hydrophobic SN60AT and 13 eyes (59.1%) in the AAB00 group. There was no statistically significant difference among the three IOL groups in the PCO development, the PCO development time and YAG laser capsulotomy rates (*P* = 0.674, *P* = 0.111, and *P* = 0.507, respectively). The PCO development time was significantly longer in the hydrophobic SN60AT than AAB00 group (*P* = 0.027). GC deposits were detected in 3 eyes (30%) in the hydrophilic SAF6125 group, 7 eyes (29.1%) in the hydrophobic SN60AT and 3 eyes (13.6%) in the AAB00 group. GC deposition and the development time of GC deposits were similar among the three IOL groups (*P* = 0.575, *P* = 0.804). At the final follow-up, BCVA was ≥ 20/40 in 41 eyes (73.2%).

**Conclusion:**

The GC deposits and PCO development were the most important problems in these eyes with hydrophilic or hydrophobic IOLs despite good visual and postoperative results. New developments are needed in terms of IOL design or content in eyes with FU.

## Introduction

Fuchs’ uveitis (FU) is characterized by chronic and low-grade inflammation of the anterior uvea accounting for approximately 2–11% of all uveitis cases [1, 2]. FU is associated with complications such as cataracts, glaucoma, and vitreous opacities. The main reasons for cataract development are chronic inflammation and long-term use of corticosteroids [2–4]. Complicated cataract occurrs in about 15–75% of FU patients, often in posterior subcapsular morphology [3]. The aim of cataract surgery in patients with FU is to visually rehabilitate the patients [3, 4].

It is known that, unlike normal eyes, an additional inflammatory reaction occurs in eyes with uveitis after cataract surgery [5, 6]. Therefore, intraocular lens (IOL) biocompatibility; its tolerance by uveal and capsular area is an important factor for the development of fewer complications. In recent years, studies have evaluated the biocompatibility of different IOL materials and designs in uveitis patients [5–9]. Cataract surgery in FU has been reported a better prognosis than other uveitis [6–11]. Nevertheless, FU has a specific feature due to vitreous involvement and chronic continuous smoldering inflammation. Therefore, the surgical outcomes of these eyes have huge importance. In light of this knowledge, we aimed to evaluate the uveal and capsular biocompatibility of the IOL, and surgical results in eyes undergoing phacoemulsification and IOL implantation due to FU-related complicated cataracts.

## Materials and methods

This retrospective study was conducted in a tertiary center Ulucanlar Eye Training and Research Hospital in Türkiye. The records of 54 FU patients who underwent phacoemulsification and foldable IOL lens implantation due to FU-related complicated cataracts were evaluated. The patients were divided into three groups according to the IOL model: hydrophilic SAF6125 (Optima fold), hydrophobic SN60AT (Alcon), and hydrophobic AAB00 (Abbott). The study followed the tenets of the Helsinki Declaration and the protocol number was approved by the Ethics Committee of the Numune Training and Research Hospital.

The diagnosis of FU was made by the same uveitis specialists (YOE, PO), based on the clinical findings including chronic, low-grade anterior chamber reaction with varying degrees of vitreous cell and fibril-like vitreous appearance, medium and/or stellate keratic precipitates in the corneal endothelium, diffuse iris atrophy, and/or heterochromia [12].

Demographic and clinical characteristics of the patients; the best-corrected visual acuity (BCVA) according to the Snellen chart, tonometry, anterior segment examination with slit-lamb biomicroscopy, and posterior segment findings were recorded at the preoperative visit and postoperative follow-ups. All patients were followed postoperatively on the 1^st^ day, 1^st^ week, 2^nd^ and 6^th^ weeks, and then at 3-month intervals.

Patients with at least 6 months of follow-up were included in the current study. Patients with a history of ocular surgery or trauma, diseases such as optic nerve diseases, diabetic retinopathy, macular degeneration, choroidal neovascularization, retinal neovascularization and/or ischemia, macular edema, other uveitic diseases and 1 > anterior chamber reaction in the last 3 months were also excluded from the study.

Intraoperative complications and postoperative complications such as uveitis activation, cystoid macular edema (CME), posterior synechia, corneal edema, IOL dislocation, IOL opacification, retinal detachment (RD) and endophthalmitis were evaluated.

Postoperative posterior capsular opacification (PCO) development and PCO development time, rate of neodymium number: YAG (Nd: YAG) laser posterior capsulotomy, giant cell (GC) deposition on the IOL, and the development time of GC deposits were recorded. Results were compared among the three groups, as well as between two hydrophobic groups (SN60AT and AAB00).

The occurrence of PCO in the slit-lamp examination was evaluated as the development time of PCO. IOL deposits have been analyzed according to Schauersberger: GCs were graded by number (0 = none; 1 = 1–9; 2 = 10–25; and 3 = > 25) and the number of small round cells was graded by density (cells/mm2) [13]. In the current study, IOL deposits were documented regarding the presence of GC deposits. We did not grade the GC deposits and small round cells. The first appearance time of GC deposits after surgery was taken as the development time of GC deposits. Activation of uveitis was considered a two-stage increase in the level of inflammation (e.g., anterior chamber cells, vitreous haze) defined according to the Standardization of Uveitis Nomenclature (SUN) Working Group guidelines) [14].

### Surgical management

All operations were performed under topical anesthesia by two surgeons (PO, YOE). Topical corticosteroids were not used before surgery. Phacoemulsification surgery was performed with the AMO WhiteStar Signature phacoemulsification system using an Ellips FX handpiece with longitudinal and elliptical tip movement. All patients underwent a 2.4 mm wide clear corneal incision, 5.0-5.5 mm diameter continuously curved capsulorhexis, and ocular viscoelastic aspiration on the back of the IOL. After uncomplicated standard phacoemulsification surgery, in-the-bag-IOL implantation (hydrophilic SAF6125 or hydrophobic SN60AT or AAB00 IOL) was performed in all eyes. All eyes were given topical dexamethasone 0.1% every 2 h and topical antibiotic (moxifloxacin 0.5%) for five times following surgery. Moxifloxacin was discontinued at 1 week and dexamethasone was used at decreasing doses for 6–8 weeks.

### Statistical analysis

The data were analyzed using IBM SPSS Statistics 22.0 (IBM, Armonk, NY, USA). Descriptive statistics were presented as mean ± standard deviation (minimum-maximum), frequency distribution, and percentage. The distribution of measurement variables was analyzed using the Shapiro-Wilk normality test. It was seen that the distributions were not suitable for normality. Frequency and percentage comparison of categorical variables were performed using the Chi-square test, comparison of pairwise means Mann-Whitney U test, and comparison of more than two means Kruskal-Wallis test. The statistical significance level was accepted as *P* < 0.05. The power analysis was performed for this study using the G*Power version 3.1.9.7 program. We found that the effect size of the study is:0.06, and α:0.05.1-β (power):0.80.

## Results

Fifty-six eyes of 55 patients were included in the study. Thirty-one (56.4%) were female, and 24 were male (43.6%). The mean age of the patients was 36.8 ± 9.4 (18–60) years. FU was unilateral in 49 patients (87.5%) and bilateral in 7 patients (12.5%). The mean postoperative follow-up period of the patients was 34.1 ± 30.1 (6-144) months. The initial clinical characteristics, preoperative and postoperative 6^th^ -month BCVA of the patients are summarized in Table 1.

**Table 1:** Clinical characteristics of the patients

	n(%)
Keratic prespitates	
Medium size KPs	49 (87.5)
Stellate / medium size KPs	3 (5.4)
Keratic prespitate place	
Whole cornea	41 (73.2)
Lower half of the cornea	11 (19.6)
Iris heterochromia	19 (33.9)
Iris nodules	
Koeppe nodules	11 (19.6)
Koeppe/Busacca nodules	2 (3.6)
The mean IOP	18.9 ± 6.5 mmHg (12-20)
Cataract	
Posterior subcapsular	50 (89.3)
Mature	6 (10.7)
Preoperative Visual Acuity	
<20/200	20 (35.8)
20/200-20/50	18 (32.1)
≥20/40	18 (32.1)
Postoperative 6^th^ month Visual Acuity	
<20/200	8 (14.3)
20/200-20/50	5 (8.9)
≥20/40	43 (76.8)

*N* Number of eyes; % Column percentage, *KPs* Keratic prespitates, *IOP* Intraocular pressure

Posterior subcapsular cataract (PSC) was observed in 50 eyes (89.3%) and mature cataracts in 6 eyes (10.7%). Fifty-four patients (98.2%) were operated on unilaterally and 1 patient (1.8%) bilaterally. Intraoperative complications such as posterior capsule rupture / anterior vitrectomy were not developed in any patient. Amsler sign which developed due to hypotonia after the first paracentesis at the beginning of phacoemulsification surgery was observed in 8 eyes (14.3%). This bleeding did not prevent visualization during surgery. None of the patients had to bleed again during other stages of the surgery.

A hydrophilic SAF6125 IOL was implanted in 10 eyes (17.9%), a hydrophobic SN60AT IOL in 24 eyes (42.8%), and a hydrophobic AAB00 IOL in 22 eyes (39.3%). Uveitis activation, corneal edema, IOL dislocation, IOL opacification, or endophthalmitis were not observed in any patient in the first 6 weeks after the surgery and subsequent follow-ups (at 3-month intervals). Six eyes (10.7%) had increased intraocular pressure that was controlled by topical 2% dorzolomid-0.5% timolol maleate in the first week after the phacoemulsification surgery. CME which developed in 2 eyes (3.6%) regressed in the first month of follow-up with posterior subtenon triamcinolone 40 mg injection in 1 eye (1.8%) and intravitreal dexamethasone implant in 1 eye (1.8%). Rhegmatogenous RD developed in 1 eye (1.8%) in the second-year follow-up.

**Fig. 1:**
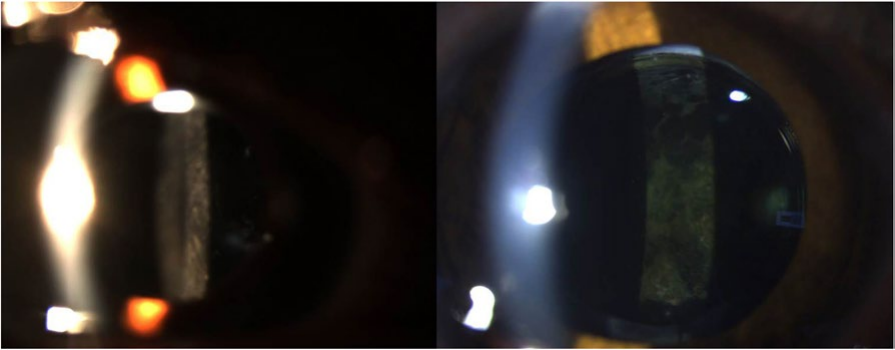
Postoperative posterior capsule opacification is shown

The PCO development was observed in a totally of 37 eyes (66.1%): these were 7 eyes (12.5%) in the hydrophilic SAF6125 IOL, 17 eyes (30.3%) in the hydrophobic SN60AT and 13 eyes (23.2%) in the hydrophobic AAB00 group (Fig. 1). There is no statistically significant difference among the three IOL groups regarding PCO development (*P* = 0.674). Also, the PCO development was similar between hydrophobic SN60AT and AAB00 IOLs (*P* = 0.250). when two hydrophobic groups were compared.

The mean PCO development time was 18.8 ± 8.5 (0–48) months in the hydrophilic SAF6125 IOL, 9.4 ± 2.8 (0–48) months in the hydrophobic SN60AT and 3.3 ± 0.8 (0–12) months in the AAB00 group. Although the mean PCO development time was longer in the hydrophilic SAF6125 group, there was no significant difference among the three IOL groups (*P* = 0.111). The mean PCO development time was significantly longer in the hydrophobic SN60AT than the AAB00 IOL group (*P* = 0.027) when two hydrophobic groups were compared. YAG laser capsulotomy was performed in 33 eyes (58.9%): 7 eyes (12.5%) were in the hydrophilic SAF6125 IOL group, 15 eyes (26.8%) in the hydrophobic SN60AT and 11 eyes (19.6%) in the AAB00 group. There was no significant difference, YAG laser capsulotomy rates were similar among the three IOLs (*P* = 0.507). It was similar between hydrophobic SN60AT and AAB00 groups (*P* = 0.251). In addition, Elsching-like opacity was not observed in any eye.

**Fig. 2:**
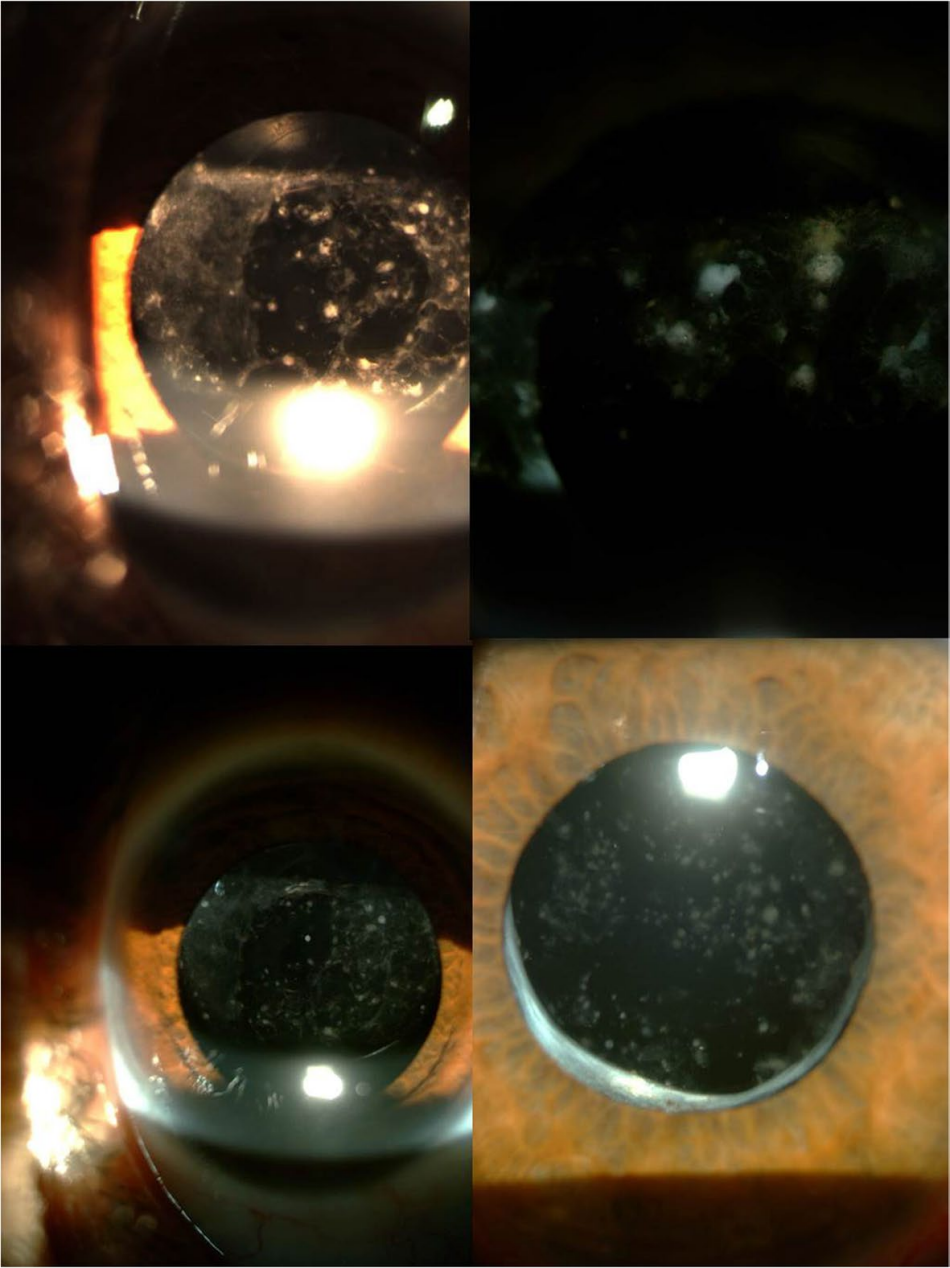
Varying degrees of giant cells accumulation on the intraocular lens are shown

The GC deposits on the IOL were detected in totally of 13 eyes (23.2%) (Fig. 2): 3 eyes (5.4%) were in the hydrophilic SAF6125 group, 7 eyes (12.5%) in the hydrophobic SN60AT and 3 eyes (5.4%) in the AAB00 group. There was no statistically significant difference among the three IOLs (*P* = 0.575). Also, the GC deposition on the IOL was similar between hydrophobic SN60AT and AAB00 groups (*P* = 0.202). The mean development time of GC deposits was 15.0 ± 9.0 (6–24) months in the hydrophilic SAF6125 group, 9.0 ± 3.05 (4–24) months in the hydrophobic SN60AT and 13.7 ± 5.9 (3–24) months in the AAB00 group. It was found to be similar among the three IOL groups (*P* = 0.804). Although the mean development time of GC deposits was shorter in the hydrophobic SN60AT group, there was no statistically significant difference between the hydrophobic SN60AT and AAB00 groups. (*P* = 0.573). Pigment deposition was not observed in any eyes. Table 2 shows the distribution of postoperative findings among the groups.

**Table 2:** Distribution of postoperative findings of IOL groups

Intraocular lens model	Hydrophilic SAF6125 IOL (*n*=10)	Hydrophobic SN60AT IOL (*n*=24)	Hydrophobic AAB00 IOL (*n*=22)	*P*-Value
**PCO incidence (n=37)**	7	17	13	0.674^a^ 0.250^a*^
**PCO development time (months) mean ± SD**	18.8 ±8.5	9.4±2.8	3.3±0.8	0.111^b^ **0.027**^c*^
**YAG laser capsulotomy (n=33)**	7	15	11	0.507^a^ 0.251^a*^
**GC deposits on IOL (n=13)**	3	7	3	0.575^a^ 0.202^a*^
**Develpoment time of GC deposits on IOL (months) mean ± SD**	15.0±9.0	9.0±3.05	13.7±5.9	0.804^b^ 0.573^c*^

*n* Number of eyes, *SD* Standard deviation, *PCO* Posterior Capsule Opacification, *IOL* Intraocular Lens, *GC* Giant Cell

^a^Chi-square test

^b^Kruskal- Wallis test

^c^Mann-Whitney U test

^*^Comparison of SN60AT and AAB00 Hydrophobic Acrylic IOLs

Preoperative BCVA was <20/200 in 20 eyes (35.8%), 20/200 − 20/50 in 18 eyes (32.1%), and ≥ 20/40 in 18 eyes (32.1%). Postoperative 6^th^ -month BCVA was <20/200 in 8 eyes (14.3%), 20/200 − 20/50 in 5 eyes (8.9%), and ≥ 20/40 in 43 eyes (76.8%). At the final follow-up, BCVA was  <20/200 in 9 eyes (16.1%), 20/200 − 20/50 in 6 eyes (10.7%), and ≥ 20/40 in 41 eyes (73.2%). Visual improvement could not be obtained due to in 4 eyes (7.1%) with glaucomatous optic atrophy, in 3 eyes (5.3%) with vitreous condensation, and in 1 eye (1.7%) with inactive macular toxoplasma scar. In addition, RD developed in 1 eye (1.7%) in the second-year follow-up.

## Discussion

In the present study, we evaluated the GCs accumulation on the IOL with biomicroscope as uveal biocompatibility and the PCO development as capsular biocompatibility and also, reported postsurgical results of hydrophilic and hydrophobic IOLs in eyes with FU. No difference was found in uveal and capsular biocompatibility among hydrophilic SAF6125, hydrophobic SN60AT and AAB00 IOL groups. Visual improvement was achieved in most of the patients (77%) after cataract surgery. Although it has been reported that the visual and post-surgical results of FU are favorable, cataract surgery outcomes and especially the formation of IOL deposits in eyes with FU remain inconclusive. On the other hand, most studies had no homogeneity in terms of IOL diversity and analysis of the specific surgical outcome for FU [5–11, 15–17]. To the best of our knowledge, our study is the only detailed study examining surgical outcomes of FU by using today’s surgical technology.

Cataract represents the most common complication in eyes with FU and it has been reported to occur at high rates up to 90.7% [4, 18]. Phacoemulsification and foldable IOL implantation are the most commonly applied surgical treatment in patients with uveitis. The choice of the right IOL material and design also plays an important role in the outcomes of cataract surgery. Several studies to date have investigated the biocompatibility of different IOL materials and designs in patients with uveitis [5–9, 15, 19]. The biological impact of an implanted IOL is at the uveal and capsular levels.

In the clinical evaluation of the capsular biocompatibility of the IOL, PCO and anterior capsule fibrosis resulting from the proliferation and migration of lens epithelial cells or the progression of lens epithelial cells to the anterior surface of the IOL are considered determinative. While no difference between hydrophobic and hydrophilic IOLs was found in some publications, others reported higher PCO rates in hydrophilic IOLs. These different results are due to the combination of various factors such as the lens material and the lens design. The optical edge design of the lens, besides the IOL material, is also considered to be important in PCO formation. The posterior sharp edge prevents the progression of lens epithelial cells on the posterior capsule with its barrier effect [5, 15, 20]. Previous studies reported the prevalence of PCO after cataract surgery of uveitic eyes to range from 34.2 to 81.7% with Nd: YAG capsulotomy rates ranging from 3.6 to 32.2% [21–23]. Rauz et al. have reported the results after phacoemulsification and implantation of various foldable IOLs in patients with uveitis. It has been reported that the development of PCO occurred in 49 eyes (81.7%) and there was no association between PCO and the various lens biomaterials, but there is no specific outcome analysis for FU in this study [5]. In our study, PCO development was observed in 66% of the total eyes, and the Nd: YAG capsulotomy rate was 60%. No significant difference was found in terms of PCO development and YAG laser capsulotomy among our study groups. Interestingly, the mean PCO development time was shorter in the hydrophobic IOL than in the hydrophilic group. Conversely, the mean PCO development time was significantly longer in the hydrophobic SN60AT group than in the AAB00 group.

Uveal biocompatibility is evaluated based on the aqueous flare and cellular accumulation on the IOL. These accumulations have two types of cellular responses. The first cellular response is the small round cell type inflammatory cell accumulation on the IOL and is seen in the early period. While the reason for this uncharacteristic trend of small cell deposition remains unclear, it has been suggested to be a sign of an ongoing active reaction. This ongoing reaction decreases over time, possibly due to the gradual recovery of the blood-aqueous barrier. The second type of cellular response, involving GCs, predominates in the late period and is considered to indicate prolonged inflammation being highly responsible for the pathogenesis of uveal biocompatibility of the IOL material. GCs, being differentiated from macrophages, are formed by epithelioid cells [24, 25]. Javadi et al., in their FU-related cataract surgery study, implanted PMMA in 32 eyes and acrylic lens (6 hydrophilic-3 hydrophobic) in 9 eyes, and IOL deposits were reported as pigment deposition in 11 eyes (26.8%), comprising mostly of patients with PMMA and the elaboration of the deposits were not performed clearly [16]. Tejwani et al. compared PMMA and acrylic lenses after cataract surgery in 103 FU-associated cataract cases and no serious inflammatory reaction was noted in any of the patients. They reported that 18 eyes (17.4%) had mild anterior chamber reaction using biomicroscopic evaluation at 5 weeks. In this study, there was no report about IOL deposits and no clear information about the details of IOL characteristics [17].

In the studies by Formanek, uveal-capsular biocompatibility and surface assessment of IOLs were evaluated [7, 15, 19, 26, 27]. In a study by Formanek in 2002, the biocompatibility of IOL variants [(hydrophilic-Hydroview® (Bausch &Lomb) (23 eyes), hydrophobic-AcrySof® (Alcon) (22 eyes) and silicone-CeeOn®-911, Pharmacia (23 eyes) IOLs)] was evaluated after phacoemulsification in uveitic and control eyes, there were 72 eyes with uveitis (only 2 eyes had FU) and 68 control eyes. In this study, it was demonstrated that GCs accumulate more in hydrophobic AcrySof IOL than in hydrophilic and silicone IOLs at 6 months after surgery. In terms of PCO, the Hydroview group had more severe PCO than the AcrySof and the CeeOn 911 groups in uveitis eyes [15]. In another study by Formanek, regarding the long-term comparison of newer version IOLs, the author suggested modern hydrophilic acrylic IOLs have better uveal biocompatibility outcomes than the hydrophobic acrylic IOL, particularly in the early postoperative period; whereas the inhomogeneity of uveitis groups was a limitation to that study and the eyes with FU were not included in this study [27].

In our study, albeit not significant the number of eyes with GCs on the IOL surface was higher in the hydrophobic SN60AT IOL group compared to the other IOLs. The development of GCs was in the late postoperative period and the meantime of GCs deposition was similar among three IOLs but this time was late in the hydrophilic SAF6125 group. The pigment deposition was observed in none of the eyes. It is difficult to compare our results with the above-mentioned studies because the number of eyes with FU is low and there is no homogeneity in terms of IOL diversity in most studies.

The optimal IOL material is a matter of debate. Linnola et al. and Johnston et al. reported the AcrySof IOLs to have a significantly higher level of fibronectin adhered to the surface than silicone, PMMA, and hydrogel lenses [20, 28]. In their studies, Formanek et al. and Roesel et al. reported significantly more GCs on hydrophobic AcrySof IOLs [15, 19, 29]. In our study, albeit not significant there was a tendency for a more GC accumulation in the Alcon group (SN60AT IOL) of hydrophobic lenses. The reason for the higher deposition of GCs on the AcrySof lens is unclear. The features of IOL material and increased and/or changed immunologic environment of uveitic eyes have been considered among the potential responsible factors. Accordingly, the individual surface molecular contents of IOLs, besides their general classification as hydrophilic to hydrophobic, seem to affect the uveal biocompatibility. In our study, we thought that the higher formation of GCs and PCO in the hydrophobic IOL than in the hydrophilic IOL is more associated with chronic continued inflammation and individual surface molecular contents rather than general classification as hydrophilic to hydrophobic.

Studies have reported good visual results after cataract surgery in patients with FU [16, 17, 30]. Tejwani et al. reported that both the postoperative 5^th^ -week BCVA (88%) and the final follow-up BCVA (93%) of the FU patients were significantly better than preoperatively [17]. In our study, preoperative BCVA was < 20/40 in approximately 68% of the patients and postoperative 6^th^-month BCVA was ≥ 20/40 in 77% of the patients.

In two different studies, complications after phacoemulsification surgery in patients with FU, except for PCO and IOL deposits, were reported as the most common anterior chamber inflammation, CME, and transient IOP elevation, but other complications such as posterior synechia, glaucoma, and RD were not reported [16, 17]. Postoperative CME (3.6%) and IOP elevation (10.7%) were among the important postoperative complications in our study. CME was treated with intraocular and periocular steroid injections, and IOP elevation was controlled with topical drops.

In the current study, favorable visual and clinical outcomes were obtained in eyes with FU, except for GC accumulation and PCO development after cataract surgery. The limitation of the study is that the anterior chamber flare meter and round cell assessment were not evaluated.

In conclusion, the GC deposits and PCO development were the most important postoperative problems in these eyes with hydrophilic or hydrophobic IOLs. Although there are new developments regarding IOL design or content, the process may progress differently in eyes with uveitis. These problems, which develop in eyes with continuous and ongoing inflammation such as FU, may mask good visual results. It is obvious that further studies on the development and the use of optimal IOLs for eyes with uveitis are needed.

## Data Availability

The datasets used and analyzed during this study are available from the corresponding author upon reasonable request.
